# Supplementary material for: Defining the nutritional input for genome-scale metabolic models: A roadmap
Source: PLoS One. 2020 Aug 14;15(8):e0236890. doi: 10.1371/journal.pone.0236890 (PMC7428157; doi:10.1371/journal.pone.0236890)
Supplement: Attachment — Submitted filename: ref_p1.pdf [file pone.0236890.s002.pdf]

Referee report

"Defining the nutritional input for genome-scale metabolic models: a roadmap"

by G. Marinos et al.

In this draft, G. Marinos et al. address the problem of modeling complex, possibly undefined nutritional media for their use as input for constraint based models of metabolic networks. They propose a guideline based on 6 steps to translate computationally the nutritional information for the model and show its application to the test case of the Lysogeny broth for *E. Coli*, where they found it improved much more the plausibility of the FBA solution both in terms of matching experimental growth physiology and overall analysis on supplementation. Although it lacks a bit of generality, the article gives an original (to the best of my knowledge) contribution towards more rigorous applications of metabolic computational modeling and thus shall deserve publication in PLOS ONE but I would suggest the author to afford the following concerns:

- It is pointed out by the authors that their aim is to make metabolic modeling more rigorous and quantitative, but then the authors apply and discuss their framework to FBA optimal solutions based on objective functions.

  The FBA approach is a very useful and wide spread approach eg to provide qualitative estimates of gene KO and/or medium growth feasibility but it misses sistematically even simple phenotypic (not even quantitative) predictions. For instance even for ideal *E. coli* cultures in minimal M9 medium (where the problem raised by the authors shall not be present) FBA fails to account for low (Journal of Biological Chemistry 278.47 (2003): 46446-46451) and high (Microbiol. Mol. Biol. Rev. 69.1 (2005): 12-50) growth rate phenotypes (as well as intermediate in non-linear way: Appl. Environ. Microbiol. 72.2 (2006): 1164-1172). In the range of growth rate explored by the authors there shall be acetate excretion, even in rich medium (Nature 528.7580 (2015): 99-104.). Is this simple prediction verified in the authors' framework? My general question is then why the authors limit themself to FBA? In principle their framework is more general and could be more useful in more quantitative computational modeling approaches, from dynamical kinetic models to Metabolic Flux Analysis. Although an application to these more quantitative approaches would add substantial value to their work, I invite the authors to at least acknowledge the limitations of their approach within the context of FBA.

- A related issue is: how does the approach applies to smaller networks, like the core metabolism of the model iJO1366? The latter are the networks usually used for truly quantitative approaches like metabolic flux analysis. Probably the issue is non trivial since one shall reliably estimate precursors concentrations.

- The authors could spend few words more in the discussion on applications to eucaryotic growth media, that are the most complex and for which the impact of their framework could be bigger.

- Minors
  line 62: ions → inorganic ions

line 63: prediction of biomass production → estimation of maximal biomass production
line 71: the accuracy of FBA ...depends.... → I would add that the accuracy on FBA could depend, most crucially, on the extent that cell metabolism is well described by linear objective functions.
line 329-332 *"When the nutritional input of the models is defined as molecular quantities and not as fluxes the lower bound of each exchange reaction can be filled with the respective concentration of the metabolite"*. I find this unclear. How is the conversion made? That in principle depends in non trivial way on the mechanism of uptake that could be different for each compound, with different kinetic constants, etc (eg Monod equation for glucose).
